# Strength and leaching characteristics of stabilized ionic rare earth tailings by microbially induced calcium carbonate precipitation

**DOI:** 10.1371/journal.pone.0347415

**Published:** 2026-05-12

**Authors:** Zhongqun Guo, Xi Cao, Qiangqiang Liu, Shaojun Xie, Yukun Zhong

**Affiliations:** 1 School of Civil Engineering and Surveying & Mapping Engineering, Jiangxi University of Science and Technology, Ganzhou, China; 2 National Engineering Research Center for Ionic Rare Earth, Ganzhou, China; CarbMin Tech, UNITED KINGDOM OF GREAT BRITAIN AND NORTHERN IRELAND

## Abstract

Ion-adsorbed rare earth mining generates extensive tailings sites with compromised structural integrity and heavy metal contamination. This study employed microbially induced carbonate precipitation (MICP) with *Sporosarcina pasteurii* to solidify and stabilize ion-adsorbed rare-earth tailings. The effects of treatment method, cementation solution (CS) concentration, and heavy metal contamination level on strength characteristics and heavy metal leaching behavior were investigated. Results showed that the unconfined compressive strength (UCS) of samples treated by cyclic grout immersion correlated positively with calcium carbonate (CaCO_3_) content, reaching a maximum of 770 kPa at 1.00 mol/L CS. Surface CaCO_3_ content increased with heavy metal concentration, while average CaCO_3_ content initially increased, then decreased with rising CS concentration. MICP treatment significantly reduced heavy metal leaching. For tailings with initial heavy metal concentrations below 500 mg/kg, leaching concentrations were suppressed below 5 mg/L across all CS concentrations tested. Microstructural analysis revealed that amorphous aggregates composed of calcite, heavy metals, and carbonate coprecipitates served as the primary cementing phase, with heavy metal stabilization achieved through precipitation, adsorption, and CaCO_3_ encapsulation. These findings demonstrate that MICP technology effectively enhances the mechanical strength of rare earth tailings while immobilizing heavy metals, offering a promising approach for tailings remediation. Further research is needed to address scalability, long-term durability, and performance variability across different tailings types.

## 1. Introduction

**Fig 1:**
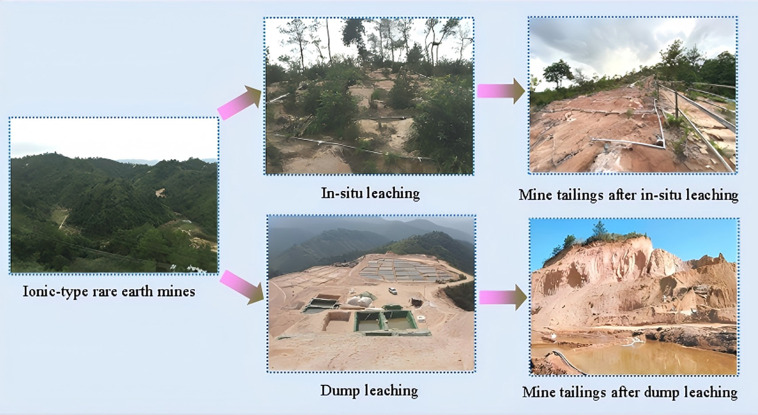
The formation process of tailings from rare earth mines.

Ion-adsorption rare earth deposits represent a globally significant strategic mineral resource. Rich in medium and heavy rare earth elements, they are widely used in critical fields such as aerospace, national defense, healthcare, new energy technologies, and advanced materials. As such, they constitute an indispensable strategic resource for China [1,2]. Their extraction technologies have undergone three iterations: tank leaching, heap leaching, and in situ leaching [3–5]. Currently, in-situ leaching is widely recommended [6,7]. However, the chemical leaching process triggers the release and migration of heavy metals from the ore/soil, leading to a reduction in ore body strength and generating large amounts of rare earth tailings (as shown in Fig 1) [8–10]. Tailings areas typically exhibit loose soil physical structure, low clay and nutrient content, and poor water retention capacity. These soil properties hinder vegetation growth and increase the risk of soil erosion and water loss within the tailings areas. Therefore, it is imperative to adopt scientific and eco-friendly solidification/stabilization techniques to properly address the potential environmental risks posed by ion-adsorption rare earth tailings.

Solidified/stabilized rare earth tailings can serve as roadbed materials, non-fired bricks, or cement admixtures, reducing the construction industry’s dependence on natural resources. The comprehensive utilization of rare earth tailings facilitates resource recovery from solid waste, decreases land occupation, and mitigates the long-term land-use conflicts caused by tailings ponds. Research on rare earth tailings solidification/stabilization represents an environmentally, economically, and socially beneficial initiative that not only addresses pollution but also redefines waste as a valuable resource, significantly advancing green mining transformation and carbon neutrality goals. Current research remains limited, primarily focusing on the development of low-cost, high-efficiency stabilizing agents [11].

Microbially induced carbonate precipitation (MICP) is an environmentally friendly and green microbial stabilization technology [12]. It can effectively bind and solidify different types of soils, thereby enhancing their strength and stability [13–15]. Liu et al.[16,17] reviewed the engineering applications of this technology and concluded that it has significant effects on soil stabilization. Héctor et al. [18] evaluated the potential of MICP to improve soil resistance to wind erosion. Their study demonstrated that biocementation can effectively enhance soil surface strength and mitigate wind erosion. Zhang et al. [19] conducted confined compressive strength tests and unconsolidated undrained shear tests on soil samples before and after MICP treatment, revealing that microbially reinforced soil demonstrated a marked increase in shear stress, confined compressive strength, and foundation bearing capacity. MICP technology can also stabilize heavy metal-contaminated soil, thereby mitigating environmental contamination [20–22]. Achal et al. [23] used the bacterium Knoellia flava CRI to induce mineral deposition for remediating lead-contaminated soil, finding that exchangeable lead was sequestered in carbonate-bound forms, thereby significantly reducing its concentration in the soil. Kumar et al. [24] found that MICP bacteria without heavy metal resistance can remediate multiple heavy metal contaminants through precipitation, further demonstrating the significant potential of MICP in heavy metal remediation. Selected bacterial strains exhibited superior stabilization performance for various heavy metals, achieving removal rates exceeding 98% within 2 hours. Concurrently, Ji et al. utilized the bacterium *Sporosarcina ureilytica* ML-2 to mitigate Pb, Cd, and Zn contamination in urban heavy metal-polluted soil. After MICP treatment, the exchangeable fractions of Cd and Zn decreased. In contrast, the carbonate-bound fractions of Pb, Cd, and Zn increased, confirming the feasibility of MICP for remediating heavy metal contamination in soil [25]. Furthermore, several scholars have researched the application of MICP technology in tailings remediation [26–28]. For instance, Héctor et al. [29] found that Sporosarcina pasteurii, through continuous urease production throughout its life cycle, catalyzes urea hydrolysis and facilitates its binding to calcium ions. The resulting carbonate mineral crystallization fills pores and cements particles within tailings, thereby reducing gas permeability and hydraulic conductivity while enhancing soil strength. Jiang et al. [30] performed solidification/stabilization remediation experiments on cadmium-contaminated tailings using *Sporosarcina pasteurii*. Their findings revealed that both negatively charged bacteria and carbonate ions can adsorb and encapsulate heavy metal ions, transforming freely dispersed heavy metals in tailings into insoluble solid-phase minerals that immobilize contaminants. Lu et al. [31] further investigated the influence of sample preparation and grouting technologies on MICP-reinforced tailings, demonstrating that mixing methods achieve more uniform bacterial distribution and superior reinforcement compared to traditional grouting, particularly in fine-grained tailings, where bacterial adsorption by fine particles limits penetration. Current research preliminarily confirms that MICP technology not only enhances the structural strength of ore bodies but also stabilizes heavy metal elements in soil. These properties demonstrate the significant applicability of MICP technology for remediating ion-adsorbed rare earth tailings. Presently, research on MICP technology for solidifying and stabilizing ion-adsorbed rare earth tailings remains relatively limited. Therefore, an in-depth investigation into the applicability, intrinsic mechanisms, and efficacy characteristics of MICP technology in this field holds substantial scientific merit and practical significance.

Despite its advantages, MICP for tailings remediation faces several intrinsic limitations that must be considered. MICP efficiency is highly dependent on environmental conditions: bacterial urease activity is optimal in neutral to alkaline pH (7–9) and decreases significantly under acidic conditions; temperature variations affect bacterial growth and enzyme kinetics, with optimal performance typically around 30–37 °C; and high ionic strength, particularly the presence of heavy metal ions, can inhibit bacterial metabolism and urease activity. These constraints are particularly relevant to ion-adsorbed rare earth tailings, which are characterized by weak acidity (pH ≈ 5.59), elevated concentrations of Pb^2+^ and Zn^2+^, and complex chemical composition. Furthermore, the practical deployment of MICP faces barriers, including the uniformity of bacterial distribution in fine-grained soils, the long-term durability of bio-cementation under field conditions, and the scalability of treatment processes. Addressing these challenges requires systematic investigation of treatment parameters and their effects on MICP performance in the specific context of rare earth tailings.

This study employs MICP technology to treat ion-adsorbed rare earth tailings, using the lead-zinc mixed contaminant concentration as a variable. The treatment efficacy of two solidification approaches—mixing method versus cyclic grout immersion method—was compared, ultimately selecting the cyclic grout immersion method for continuous solidification stabilization of contaminated tailings. The study further analyzes the strength development and contaminant leaching characteristics of MICP-treated ion-adsorbed rare earth tailings, elucidating the underlying microscopic mechanisms. This research provides scientific references for addressing potential environmental risks associated with ion-adsorbed rare earth tailings.

## 2. Experimental materials

### 2.1. Soil samples

The experimental tailings soil was collected from an ion-adsorbed rare earth mining area in Fujian Province, China. Under natural conditions, the lead content in the tailings ranges from 55 mg/kg to 520 mg/kg, while the zinc ion content varies from 81 to 300 mg/kg [32]. Compared with Fujian Province’s soil heavy metal safety standards, the maximum detected levels exceeded safety thresholds by 15-fold for lead and 34-fold for zinc [33].

Soil sampling was conducted in designated excavation zones of the rare earth mining area. To mitigate errors and adverse effects of soil heterogeneity across sampling locations on MICP solidification/stabilization efficacy, the collected soil was subjected to laboratory leaching with a 6% MgSO4 solution (pH 2) as the lixiviant. After 14 days of leaching, analytical-grade Pb(NO_3_)_2_ and Zn(NO_3_)_2_ were incorporated into the soil to artificially simulate tailings generated by salt-based lixiviant extraction in ion-adsorbed rare earth mining (hereinafter referred to as rare earth tailings). The synthesized tailings were co-contaminated with Pb^2+^ and Zn^2+^, each at target concentrations of 100, 300, 500, and 700 mg/kg, respectively.

**Fig 2:**
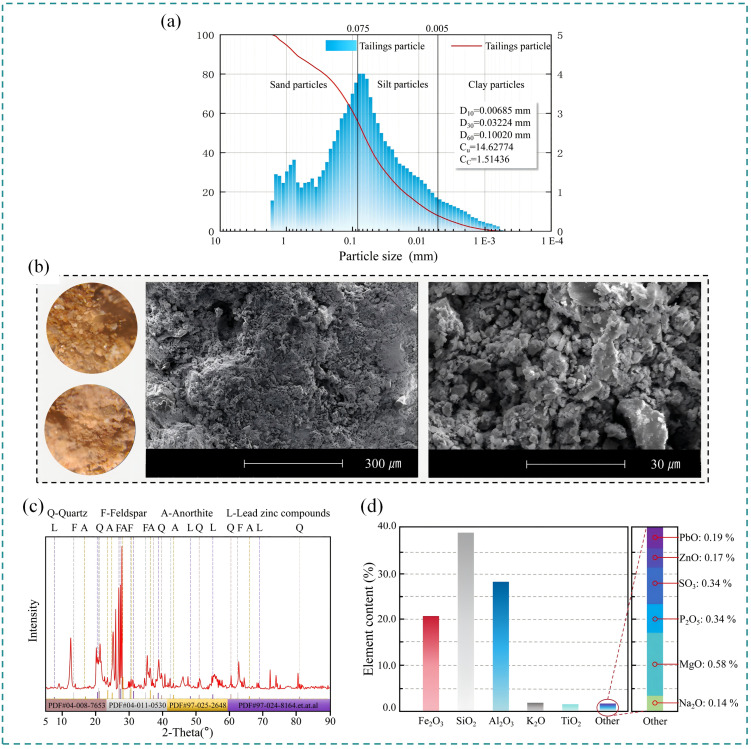
Basic Parameters of Rare Earth Tailings. **(a)** Particle size distribution curve of ionic rare earth tailings. **(b)** Scanning electron micrograph of Ionic Rare Earth Tailings. **(c)** Structural and Compositional Characterization of Ionic Rare Earth Tailings by XRD. **(d)** Structural and Compositional Characterization of Ionic Rare Earth Tailings by XRF.

According to the Chinese National Standard for Geotechnical Testing Methods (GB/T 50123-2019), some tests were conducted to determine the fundamental properties of rare earth tailings. Laser diffraction analysis (LDA) was performed using a Bettersize 3000 laser particle size analyzer (Dandong Baiter Instrument Co., LTD, Dandong, China) to characterize the particle size distribution of the tailings, with results illustrated in Fig 2a.

The primary mineral composition was determined by XRF analysis of fused glass discs using a wavelength-dispersive X-ray spectrometer (Panaco Company, Eindhoven, The Netherlands), as detailed in Table 1. Standard compaction tests, soil pH measurements, and liquid-plastic limit tests were conducted to determine the maximum dry density, optimum moisture content, and pH value of the tailings soil. The natural water content was measured using the cutting ring method, and all results are summarized in Table 2.

**Table 1:** Major chemical components of ionic rare earth tailings.

Composition	Na_2_O	MgO	Al_2_O_3_	SiO_2_	P_2_O_5_	SO_3_	K_2_O	TiO_2_	Fe_2_O_3_	ZnO	PbO
Content (%)	0.14	0.58	32.85	39.82	0.26	0.34	1.85	1.56	22.60	0.18	0.12

**Table 2:** Basic physical parameters of ionic rare earth tailings.

Water Content *ω* (%)	Density *ρ*/ (g/cm^3)^	Liquid Limit WL (%)	Plastic Limit WP (%)	Plasticity Index IP	pH Value
23.7 ± 0.1	1.72 ± 0.0	31.3 ± 0.5	18.7 ± 0.2	12.6 ± 0.1	5.59 ± 0.0

Microstructural pore characteristics of the soil samples were examined by scanning electron microscopy (SEM) using a Zeiss Sigma 500 microscope (Carl Zeiss AG, Oberkochen, Germany), as shown in Fig 2b. X-ray diffraction (XRD) patterns were acquired using a Bruker D8 Advance diffractometer (Bruker AXS GmbH, Karlsruhe, Germany), presented in Fig 2c, X-ray fluorescence (XRF) analysis was conducted using a wavelength dispersive X-Ray spectrometer (Panaco Company, Eindhoven, The Netherlands), following the fused bead method, presented in Fig 2d.

### 2.2. Bacterial cultivation and growth

**Fig 3:**
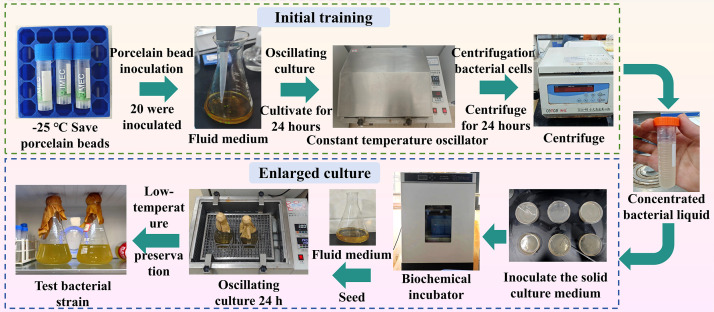
The culture process of the bacterial generation.

The bacterial strain used in the experiment was *Sporosarcina pasteurii* (deposition No. CGMCC 1.3687), obtained from the China General Microbiological Culture Collection Center (CGMCC). Following activation, the strain was cryopreserved in magnetic beads. Preliminary cultivation and scale-up propagation were conducted using liquid and solid culture media, respectively [[34], [35]], as illustrated in Fig 3.

The culture medium composition was as follows: 5.0 g peptone, 3.0 g beef extract, 20.0 g urea, and 20 g agar powder dissolved in 1 L of deionized water. After thoroughly mixing the components, the pH was adjusted to 7.0 with 1 M NaOH, then autoclaved at 121 °C for 30 min before bacterial inoculation. Cryobeads containing preserved bacteria were aseptically transferred into 250 mL of sterile liquid medium and incubated at 30 °C with orbital shaking (150 rpm) for 24 h.

**Fig 4:**
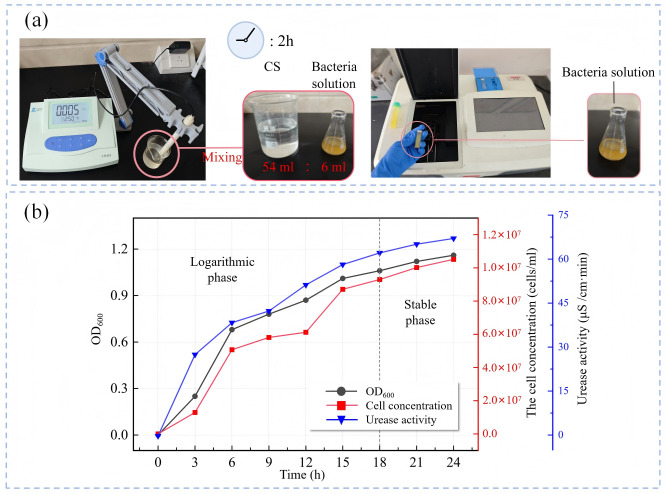
Determination of physiological and biochemical characteristics of *Sporosarcina pasteurii:* (a) Determination process, (b) Growth curve and urease activity of *Sporosarcina pasteurii*.


As illustrated in Fig 4a, during the scale-up culture process, aliquots of the bacterial suspension were sampled every 2 hours over the 0–40 hour period to determine bacterial concentration and urease activity. Bacterial concentration was quantified by measuring the optical density at 600 nm using a UV-Vis spectrophotometer, with cell counts calculated via equation (1); urease activity was assessed by monitoring conductivity changes over 5 minutes in a mixture of 6 mL bacterial suspension and 54 mL urea solution using a conductivity meter [36]. The average conductivity change per minute (μS·cm^−1^·min^1^) was computed, and Whiffin’s conversion equation (2) was applied to derive the urea hydrolysis rate per minute (μmol urea hydrolyzed·min^−1^) of the suspension. The urease activity of bacteria was thus characterized by the rate of urea hydrolysis per minute per unit volume of bacterial suspension. Physiological and biochemical characteristics of the bacteria are presented in Fig 4b. Cultured bacterial suspensions were stored at 4 °C for subsequent use [37].

$$Y=\text{8}.\text{59}\times {\text{10}}^{\text{7}}\times Z^{\text{1}.\text{3627}}$$ (1)

U=10×KA (2)

where Y represents the cell concentration (cells/ml), Z denotes the value of OD_600_, U represents the urea hydrolysis rate (μS/cm·min), K represents the rate of change in conductivity (μS·cm^−1^·s^1^), and A represents the cell constant (cm^1^).

The cementation solution, primarily composed of equimolar concentrations of urea (CO(NH_2_)_2_) and calcium chloride (CaCl_2_), was formulated to provide optimal conditions and essential nutrients for bacterial growth [38]. Consequently, the solution additionally contained NH₄Cl and beef extract. For the experiment, CS concentrations were set at 0.50, 0.75, 1.00, and 1.25 mol/L, respectively.

## 3. Experimental design

**Fig 5:**
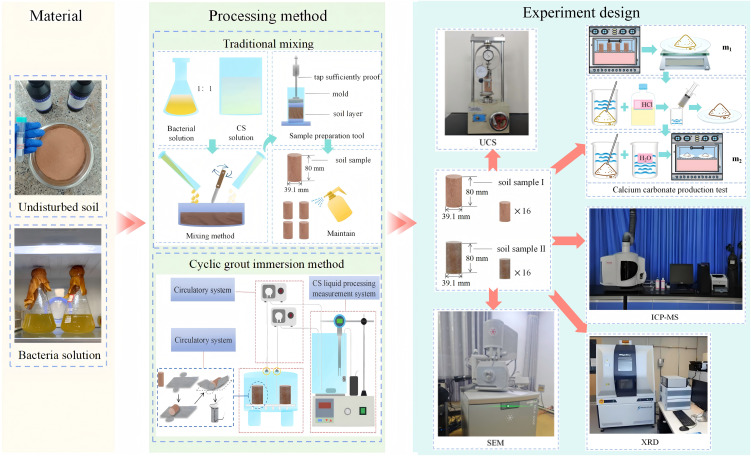
Experiment design. **(a)** YYW-Ⅱ Unconfined Compression Strength Testing Machine, **(b)** Calcium carbonate test, **(c)** Inductively Coupled Plasma Mass Spectrometer (ICP-MS), **(d)** FE-SEM, **(e)** X-ray Diffractometer.

The experimental design and testing are illustrated in Fig 5. Due to the relatively limited application of MICP technology in ion-adsorbed rare earth tailings remediation, this study selected two treatment methods—traditional mixing and the cyclic grout immersion method—to investigate the effectiveness of different MICP approaches.

Initially, solidification treatment was applied to rare earth tailings without initial heavy metals, and a comparative analysis was conducted to evaluate the curing effects of both methods. Subsequently, the more effective method was employed for the solidification and stabilization of rare earth tailings containing initial heavy metals, providing a more comprehensive analysis of the remediation effects of MICP technology on ion-adsorbed rare earth tailings. The tests were conducted in triplicate. The details of the experimental design are shown in Table 3.

**Table 3:** Experiment design.

Curing Method	Initial Heavy Metal Content (mg/kg)	CS Concentration (mol/L)	CaCO_3_ Precipitation Test	UCS Test	Toxicity Leaching Test	XRD	SEM
Traditional Mixing	0	0.5, 0.75, 1.0, 1.25	✓	✓			
Cyclic Grout Immersion Method	0	0.5, 0.75, 1.0, 1.25	✓	✓	✓	✓	✓
	100	0.5, 0.75, 1.0, 1.25	✓	✓	✓	✓	✓
	300	0.5, 0.75, 1.0, 1.25	✓	✓	✓	✓	✓
	500	0.5, 0.75, 1.0, 1.25	✓	✓	✓	✓	✓
	700	0.5, 0.75, 1.0, 1.25	✓	✓	✓	✓	✓

## 4. Testing methods

### 4.1. Microbial treatment of ion-adsorbed rare earth tailings soil

#### 4.1.1. Mixing method.

**Fig 6:**
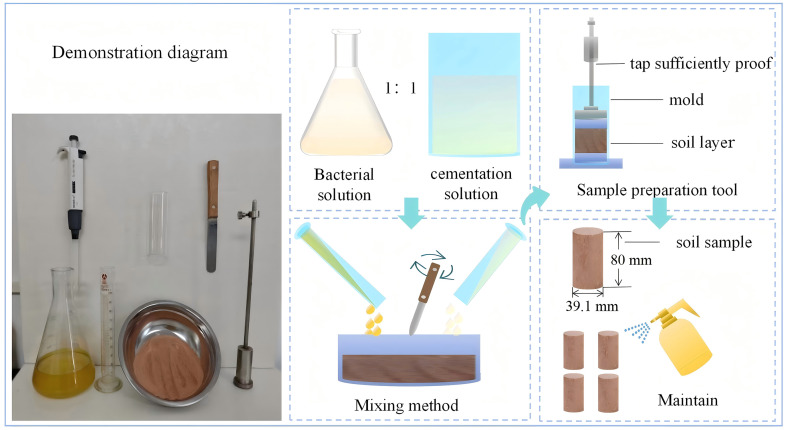
Demonstration diagram of ionic rare earth tailings soil.

For the mixing method, 20 mL of bacterial suspension (OD₆₀₀ ≈ 1.4) was mixed with 160 g of tailings and allowed to stand for 24 h to ensure bacterial attachment. An equal volume of cementation solution was then added with rapid stirring. The mixture was compacted layer by layer into molds (Ф 39.1 mm × 80 mm). After demolding, specimens were cured in a standard curing room for 7 days before testing. The procedure is illustrated in Fig 6.

#### 4.1.2. Cyclic grout immersion method.

**Fig 7:**
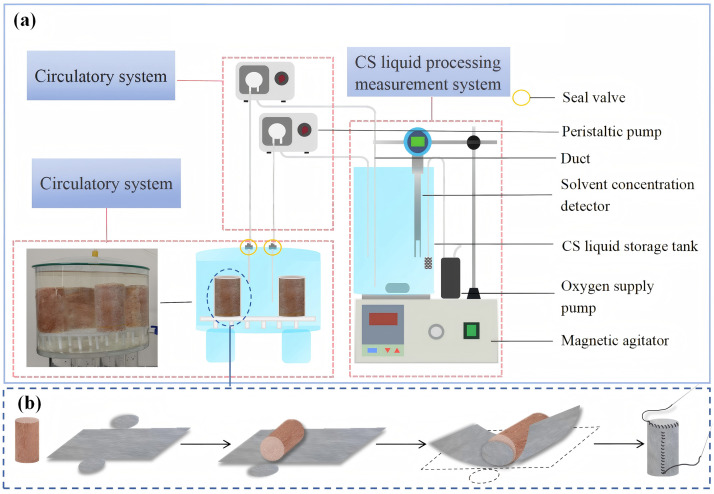
Cyclic grout immersion reactor. **(a)** Cyclic grout immersion reactor. **(b)** The step of placing the soil sample in the flexible mold.

A microbial cyclic grout immersion reactor was developed based on the integral immersion method [39–42] with modifications (Fig 7a). The apparatus comprises three systems: grouting reaction, circulation, and processing of the cementation solution. The grouting reaction system includes a reaction chamber and a sample holder. The cementation solution system consists of a reservoir equipped with a magnetic stirrer, air pump, and calcium ion detector to maintain solution homogeneity and oxygen saturation.

The key innovation is the external delivery of homogenized, oxygen-saturated cementation solution via a peristaltic pump, avoiding disruptive forces within the reaction chamber that could damage unconsolidated specimens [43, 44]. The system also enables real-time solute monitoring and replenishment, improving solution utilization efficiency.

For specimen preparation, 40 mL of bacterial suspension (OD₆₀₀ = 1.12) was mixed with tailings and compacted into molds (Ф39.1 mm × 80 mm). After demolding, specimens were placed in permeable geotextile molds (Ф39.4 mm × 83 mm, Fig 7b) and transferred to the reactor for 7 d of cyclic immersion. Cementation solution was injected at 1 mL/min, completing one cycle every 3 d. After treatment, specimens were removed and cured for 7 days before testing.

### 4.2. Unconfined compressive strength test

Unconfined compressive strength (UCS) of tailings specimens before and after MICP treatment was measured using a YYW-II unconfined pressure apparatus (Fig 5a). Specimens (Ф38.0 mm × 80.0 mm) were loaded at 1.0 mm/min until failure, and the maximum pressure was recorded. Tests were completed within 8–20 min.

### 4.3. Calcium carbonate content test

Carbonate content (including metal carbonates and CaCO_3_) was determined by hydrochloric acid leaching. After treatment, specimens were dried at 105 °C to constant weight (m₁), then immersed in 20% HCl until bubble cessation. The solution was filtered (0.45 μm), and the residues were washed with deionized water to remove soluble salts. Samples were re-dried at 105 °C to constant weight (m_2_). Carbonate content was calculated as [45]:


$$\text{CaC}{\text{O}}_{\text{3}}(\%)=\frac{\left(m_{\text{1}}-m_{\text{2}}\right)}{m_{\text{1}}}\times \text{100}\%$$ (3)

### 4.4. Toxicity characteristic leaching procedure test

As shown in Fig 5c, the heavy metal leaching behavior was evaluated using the Toxicity Characteristic Leaching Procedure (TCLP). Soil samples were dried (105 °C, 24 h) and sieved (2 mm). Then, 10 g of soil was mixed with 200 mL of extraction solution (glacial acetic acid, pH = 2.8) and shaken at 200 rpm for 18 ± 2 h. The supernatant was centrifuged (4000 rpm, 10 min) and filtered (0.45 μm). ICP-OES determined heavy metal concentrations in the filtrate. Three replicates were conducted for each condition.

### 4.5. Microstructural characterization

As shown in Fig 5d, the sample microstructure was examined using an MLA650F field emission scanning electron microscope (FE-SEM). Specimens were fixed on copper substrates with conductive adhesive, gold-sputtered to enhance conductivity, and observed under vacuum at increasing magnifications. Mineral composition was analyzed by X-ray diffraction (XRD) over 10–90° (2θ) at <5°/min, with data processed using MDI Jade 6.5 software.

### 4.6. Pearson correlation analysis

This study uses Pearson’s correlation coefficient to quantitatively analyze the relationships between various parameters of ion-adsorbed rare earth tailings before and after MICP solidification/stabilization. Pearson’s correlation coefficient is a statistical measure used to accurately assess the strength and direction of the linear relationship between two continuous variables. Based on n pairs of observed data {(xi, yi) | i = 1, 2, …, n} for variables x and y, the Pearson correlation coefficient between them is defined by the equation (4).


$$=\frac{\sum_{i=\text{1}}^{n}\left(x_{i}-\overset{―}{x})(y_{i}-\overset{―}{y}\right)}{\sqrt{\sum_{i=\text{1}}^{n}{\left(x_{i}-\overset{―}{x}\right)}^{\text{2}}}\sum_{i=\text{1}}^{n}{\left(y_{i}-\overset{―}{y}\right)}^{\text{2}}}$$ (4)

## 5. Results and discussion

### 5.1. Comparative analysis of different microbial treatments for rare earth tailings soil

**Fig 8:**
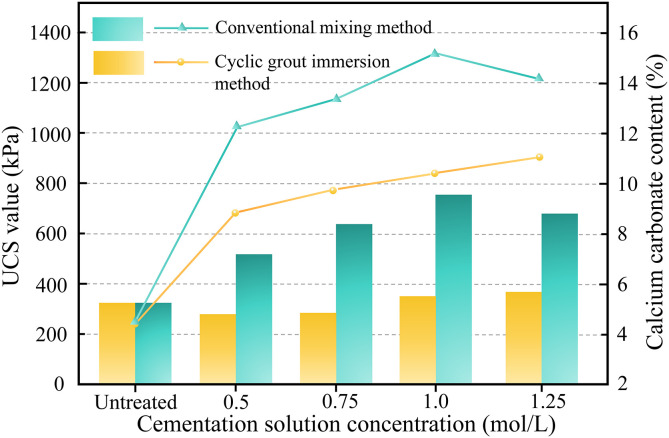
The variation trends of the unconfined compressive strength and the amount of calcium carbonate generated in the soil samples before and after the treatment of the two schemes.

This study investigates the treatment efficacy of MICP technology for solidifying ion-adsorbed rare earth tailings. Two treatment methodologies—conventional mixing and the cyclic grout immersion method—were applied to heavy metal-free rare earth tailings. Through a comparative analysis of unconfined compressive strength (UCS) and calcium carbonate precipitation before and after treatment, the optimal method demonstrating the remediation effectiveness of MICP for ion-adsorbed rare earth tailings was identified. Fig 8 presents comparative data of UCS and carbonate precipitation for specimens treated by both methods.

As illustrated in Fig 8, variations in the concentration of the cementation solution significantly affect the amount of calcium carbonate generated under both treatment schemes. When the conventional mixing method was employed, the amount of calcium carbonate in the soil samples increased gradually with increasing concentration of the cementation solution, reaching a maximum of 11.1% at 1.25 mol/L. In contrast, when the cyclic grout immersion method was employed, the amount of calcium carbonate in the soil samples initially increased and then decreased, reaching a maximum of 15.1% at a concentration of 1.00 mol/L. After treatment with the conventional mixing method, the unconfined compressive strength of the soil samples did not generally increase due to the newly generated calcium carbonate; only when the cementation solution concentration exceeded 1.00 mol/L did the strength increase, whereas at 0.50 mol/L and 0.75 mol/L, the unconfined compressive strength was lower than that of the untreated soil. The unconfined compressive strength of the soil samples treated by the cyclic grout immersion method showed a positive correlation with the amount of calcium carbonate generated, reaching a maximum value of 770 kPa at a cementation solution concentration of 1.00 mol/L.

This phenomenon occurs because lower cementation solution concentrations yield reduced calcium carbonate precipitation rates and smaller crystal sizes, primarily adhering to the surfaces of fine particles. The diminutive crystals exhibit insufficient bonding capacity for clay and silt particles, resulting in poor cementation and low unconfined compressive strength (UCS). Fu et al. [46] similarly documented limited soil solidification by *Sporosarcina pasteurii* under conditions of low-concentration cementation solutions. As the concentration of the cementation solution increases, the precipitation mechanism shifts: new carbonate preferentially deposits onto existing crystals rather than forming new nucleation sites. This process generates larger calcite crystals bridging silt and clay particles, thereby enhancing structural stability and strength [47]. These observations align with the conclusion that the concentration of the cementation solution critically regulates precipitation efficiency, crystal morphology, and distribution. In MICP, the concentration of the cementation solution serves as a key governing factor for carbonate precipitation kinetics. A critical threshold exists below which UCS improvement remains unattainable. Experimental results demonstrated that cyclic grouting produced 36% higher carbonate precipitation than conventional mixing, yielding more significant UCS enhancement without strength reduction below critical concentrations. Moreover, the cyclic grout immersion method allows for cementation solution recycling, resulting in material costs comparable to conventional mixing and offering practical advantages for potential field applications. Consequently, cyclic grouting more effectively demonstrates the remediation efficacy of MICP for ion-adsorbed rare earth tailings, warranting its exclusive adoption in subsequent experiments [48,49].

### 5.2. Temporal dynamics of carbonate precipitation

**Fig 9:**
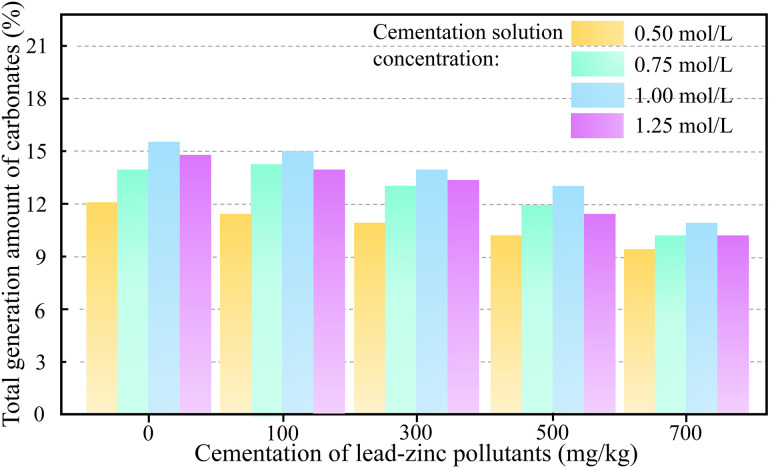
The relationship between the amount of calcium carbonate generated and the concentration of lead and zinc pollutants in the soil.

In the field of microbially mediated soil remediation, carbonate precipitation (including metal carbonates and calcium carbonate) exhibits a positive correlation with soil strength. Additionally, carbonate precipitates demonstrate significant adsorption and coprecipitation effects on heavy metals [50,51]. Consequently, carbonate yield constitutes the material basis governing soil solidification and stabilization characteristics. Fig 9 illustrates the quantitative relationship between total carbonate precipitation and lead-zinc contaminant concentrations in soil specimens.

As shown in Fig 9, increasing lead-zinc contaminant concentrations were correlated with a marked decline in total carbonate precipitation within specimens treated via the cyclic grout immersion method. When contaminant levels increased from 100 mg/kg to 700 mg/kg, specimens immersed in a 1.0 mol/L cementation solution showed an 18.0% reduction in carbonate precipitation (from 15.0% to 12.3%). This trend persisted across all concentrations of the cementation solution tested.

The phenomenon was attributed to the inhibitory effects of heavy metal ions on urease-producing microorganisms. Previous studies have elucidated the molecular mechanisms underlying this inhibition: Pb^2+^ and Zn^2+^ can penetrate cell walls and bind to essential groups (such as sulfhydryl groups) in the active sites of urease, leading to conformational changes that inhibit enzyme activity [52]. Xie et al. [53]. reported that when Pb^2+^ concentration reached 60 mM, the extracellular polymeric substances and cell membranes of *Sporosarcina pasteurii* could not effectively protect urease activity, resulting in a reduction of immobilization efficiency to below 50%. Hu et al. [54] also found that heavy metal concentrations significantly affected bacterial cell concentration and urease activity in Bacillus intermedius TSBOI, with this inhibitory effect exhibiting a clear concentration dependence. The 18.0% reduction in carbonate precipitation observed in this study aligns with the reported inhibition threshold ranges for urease-producing bacteria exposed to Pb^2+^ and Zn^2+^. Notably, even under high contamination levels of 700 mg/kg, MICP still occurred (12.3% carbonate precipitation), indicating that *Sporosarcina pasteurii* retained partial metabolic activity within this concentration range, though activity was substantially inhibited.

It is worth noting that the reduced carbonate precipitation at higher heavy metal concentrations may result from both biological and chemical mechanisms: direct toxicity of heavy metals to bacterial metabolism, and ion competition effects, where heavy metal ions compete with Ca^2+^ for available CO_3_^2−^ to form metal carbonates. The presence of PbCO_3_ and ZnCO_3_ observed in the XRD analysis (Section 5.5) confirms that ion competition indeed occurs.

When the cementation solution concentration increases from 0.5 mol/L to 1.0 mol/L, the total carbonate generation of the soil sample with a lead-zinc pollutant concentration of 100 mg/kg increases from 11.0% to 14.9%, a 1.35-fold increase. However, when the cementation solution concentration increases to 1.25 mol/L, the total carbonate generation of the soil sample decreases to 13.8%. In contrast, the total carbonate generation is highest when the concentration of the cementation solution is 1.0 mol/L. The total carbonate generation of the soil sample at other heavy metal concentrations also shows that at 1.25 mol/L, it is lower than at 1.0 mol/L. This phenomenon can be explained by the effect of urea and calcium ion concentration on the urease activity of the bacteria. The prerequisite for microbial-induced mineralization is that urease bacteria hydrolyze urea. Urease bacteria provide nucleation sites for carbonate precipitation and an alkaline environment for urea hydrolysis, so their activity directly affects the efficiency of urea hydrolysis, and thus the carbonate generation. A moderate amount of urea can stimulate urease activity, while excessive urea concentration inhibits bacterial growth. Part of the reason is that high urease expression and high catalytic intensity limit the supply of materials and energy required for bacterial growth and reproduction. As the urea concentration increases, bacterial urease expression first increases and then decreases. At the same time, the effect of calcium ion concentration is also dual. An appropriate increase in calcium ion concentration can promote bacterial growth and metabolism, which is beneficial for urease-producing bacteria to hydrolyze urea. However, excessively high concentrations of calcium ions inhibit urease activity, reducing the production of carbonates.

**Fig 10:**
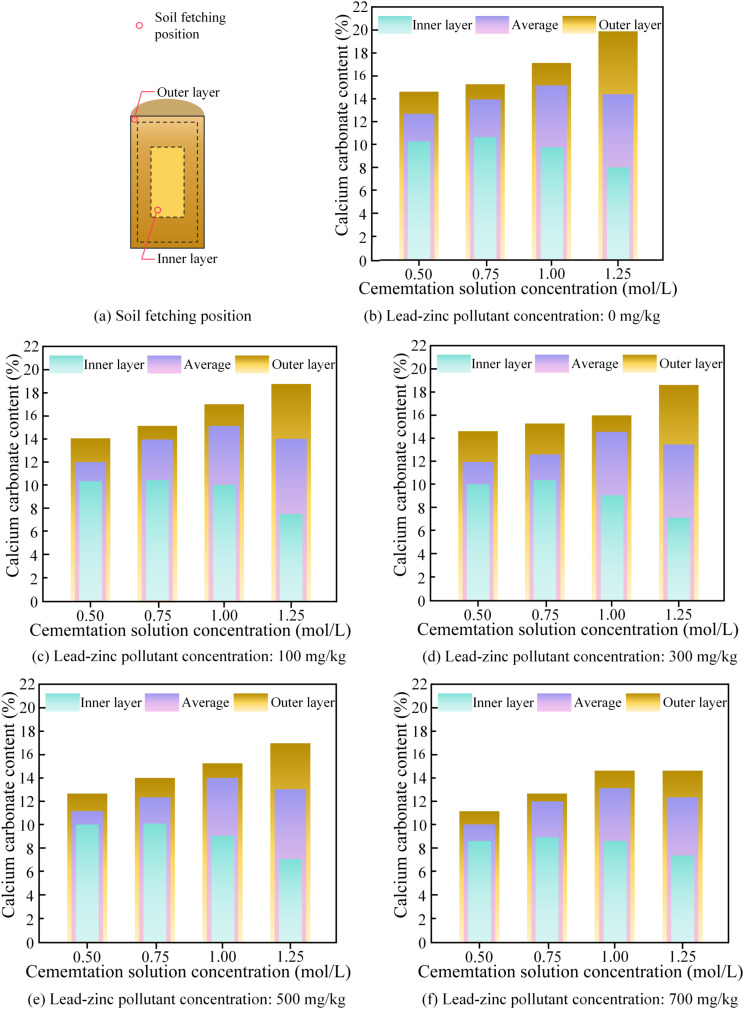
The relationship between internal and external carbonate formation and lead-zinc concentration in soil samples. **(a)** Soil fetching position; **(b)** Lead-zinc pollutant concentration: 0 mg/kg; **(c)** Lead-zinc pollutant concentration:100 mg/kg; **(d)** Lead-zinc pollutant concentration: 300 mg/kg; **(e)** Lead-zinc pollutant concentration: 500 mg/kg; **(f)** Lead-zinc pollutant concentration: 700 mg/kg.

Fig 10 illustrates the relationship between the carbonate production in the soil sample’s interior and exterior and the concentrations of lead-zinc pollutants. The observation results showed that as the concentration of the cementation solution increases, the carbonate production in the surface layer of the soil sample tends to increase, while the carbonate production in the interior decreases. The difference in the carbonate production between the interior and exterior increased significantly with the rising concentration of the cementation nutrient solution. This is because when the cementation solution concentration is at a lower level (below 1.0 mol/L), the MICP reaction is slow, with a slower rate of carbonate production. The resulting crystals are smaller and evenly distributed, which favors the subsequent penetration of the cementation solution into the interior of the soil sample, promoting carbonate production within the interior. However, when the cementation solution concentration is at a higher level (greater than 1.0 mol/L), the MICP reaction becomes more intense, leading to rapid carbonate production at the surface layer of the soil sample, which blocks the surface voids and hinders the penetration of the cementation solution, resulting in an uneven spatial distribution of the carbonate products within the soil sample, and an overall reduction in the total production.

### 5.3. Unconfined compressive strength

**Fig 11:**
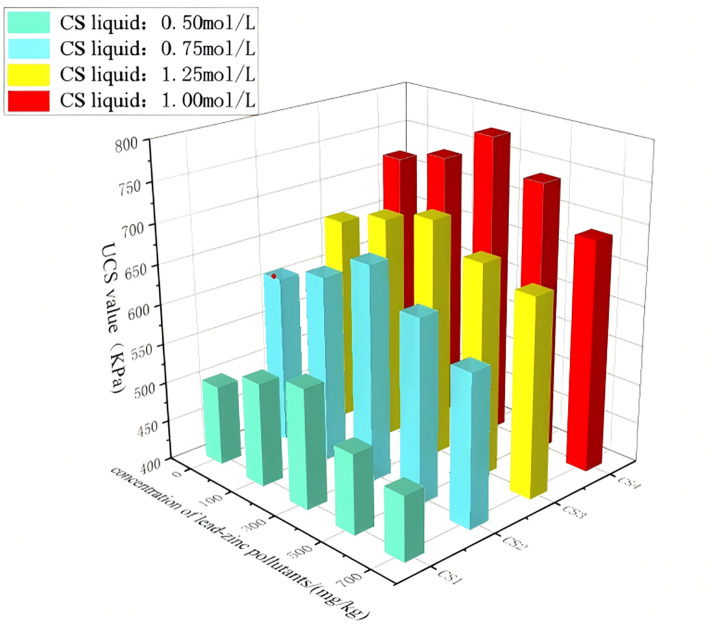
Relationship between soil samples with unlimited compressive strength and pollutant content of lead and zinc2.

Fig 11 shows the relationship between UCS and the concentration of lead-zinc pollutants. As shown in the figure, after cyclic immersion treatment, the unconfined compressive strength of the soil sample was significantly improved. Under different concentrations of the cementation solution, the unconfined compressive strength of the soil sample increases and then decreases as the concentration of lead-zinc pollutants increases. When the concentration of lead-zinc pollutants in the soil increases from 0 to 300 mg/kg, the unconfined compressive strength of the soil treated with a 1 mol/L concentration of cementation nutrient solution increases from 720 to 780 kPa. However, when the concentration of lead-zinc pollutants increases to 700 mg/kg, the unconfined compressive strength of the soil decreases to 690 kPa. The unconfined compressive strength of the soil samples under other cementation solution concentrations also follows the trend of first increasing and then decreasing as the concentration of lead-zinc pollutants increases.

This change is inconsistent with the variation in carbonate formation and lead-zinc pollutant content shown in Fig 11. This discrepancy arises because *Sporosarcina pasteurii* maintains high activity at low heavy metal concentrations, enabling effective immobilization of zinc and lead ions. Jiang et al. [55] reported that bacterial activity and urease activity were largely unaffected when the Pb^2+^ concentration was below 30 mM. Zha et al. [56] also found that trace amounts of Pb^2+^ promoted bacterial growth to some extent, whereas excessive Pb^2+^ exhibited toxicity and inhibited the MICP solidification effect. Microstructural analysis shown in Fig 14 provides direct evidence for this observation: small clusters of lead and zinc mineral crystals, along with amorphous aggregates formed by the coprecipitation of calcium carbonate and heavy metal carbonates, were observed within the soil matrix. These newly formed products effectively filled interparticle pores and bonded adjacent soil particles, acting as cementing agents that enhanced soil strength. Additionally, the insoluble solid-phase minerals formed through microbial adsorption and encapsulation possess cementing properties that enhance soil strength. Therefore, although low concentrations of heavy metal pollutants inhibit calcium carbonate formation to some extent, soil strength does not decrease accordingly. However, when the initial heavy metal concentration increases further, its biotoxicity inhibits the activity of *Sporosarcina pasteurii*, hindering its reaction with the cementation solution and reducing the amount of calcium carbonate precipitate formed, thereby diminishing its ability to fill soil pores. On the other hand, the introduction of heavy metal ions into the soil increases the content of soluble salts, which replace some soil particles that serve as the structural framework, ultimately leading to a significant reduction in soil strength.

**Fig 12:**
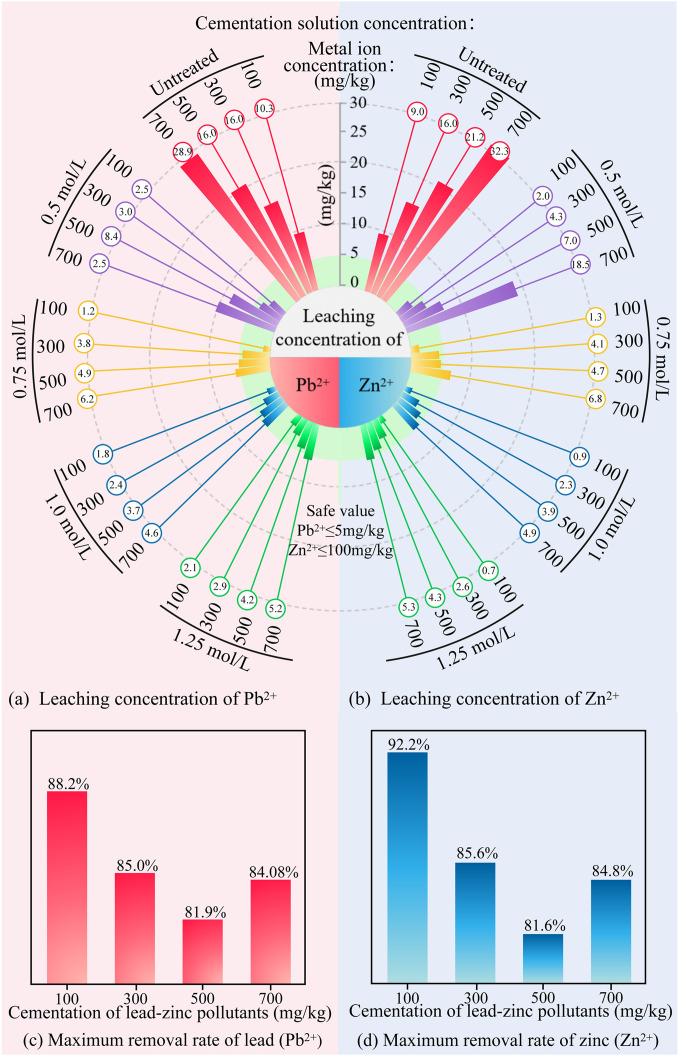
Changes in the toxicity leaching concentration of stabilized rare earth tailings under different cementation solution concentrations. **(a)** Leaching concentration of Pb^2+^; **(b)** Leaching concentration of Zn^2+^;(c) Maximum removal rate of lead (Pb^2+^); **(d)** Maximum removal rate of zinc (Zn^2+^).

**Fig 13:**
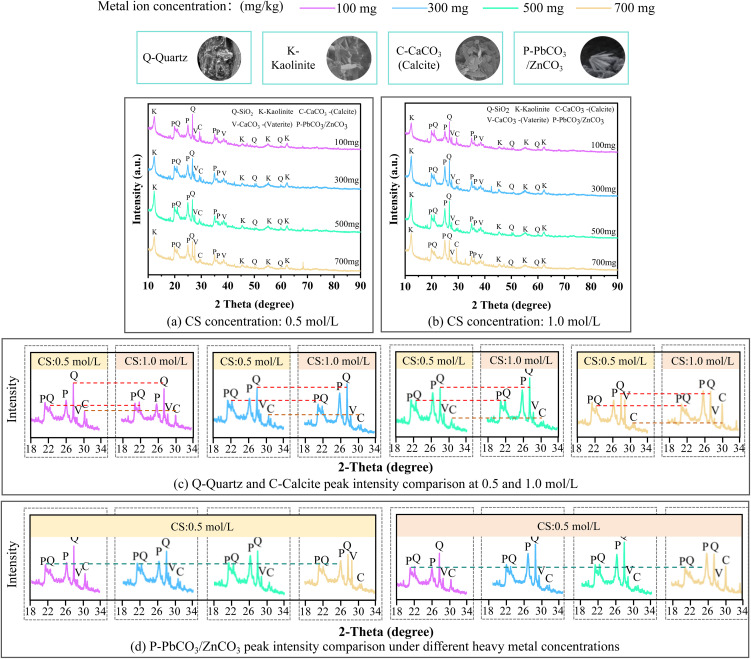
XRD patterns of biomineralization precipitation under different working conditions. **(a)** CS concentration: 0.5 mol/L, **(b)** CS concentration: 1.0 mol/L, **(c)** Q-Quartz and C-Calcite peak intensity comparison at 0.5 and 1.0 mol/L, **(d)** P-PbCO_3_/ZnCO_3_ peak intensity comparison under different heavy metal concentrations.

### 5.4. Toxic leaching characteristics

In rare earth tailing soils, heavy metal elements are highly susceptible to leaching into the surrounding environment due to the infiltration effects of rainfall, surface water, and groundwater, which can lead to contamination. Therefore, the Toxicity Characteristic Leaching Procedure (TCLP) was used to evaluate the leaching behavior of lead and zinc ions in MICP-treated rare earth tailings. Fig 12 shows the changes in toxicity leaching concentrations at different cementation solution concentrations and initial heavy metal levels.

As shown in Fig 12, the leaching concentrations of Pb^2+^ and Zn^2+^ were positively correlated with the initial heavy metal content. With increasing cementation solution concentration, the leaching concentrations generally exhibited a trend of initial decrease followed by a slight rebound. This can be attributed to the inhibitory effects of heavy metals on microbial activity: higher initial heavy metal concentrations suppress the growth and urease activity of *Sporosarcina pasteurii*, reducing urea hydrolysis efficiency [57] and consequently diminishing carbonate precipitation and heavy metal immobilization. Similar inhibitory effects have been reported in other tailings systems. Lu et al. [58] found that Cu^2+^ significantly inhibited the growth and urease activity of Sporosarcina pasteurii in copper tailings, consistent with our observation that higher heavy metal concentrations lead to reduced MICP efficiency.

The stabilization effect was evaluated based on the limits specified in the Chinese standard GB 5085.3—2007 (Pb^2+^ ≤ 5 mg/L, Zn^2+^ ≤ 100 mg/L). For tailings with initial heavy metal content below 500 mg/kg, microbial treatment successfully controlled the leaching concentrations of both Pb^2+^ and Zn^2+^ below these regulatory limits across most tested conditions. For tailings with 700 mg/kg initial heavy metal content, Zn^2+^ leaching was controlled below 100 mg/L, while Pb^2+^ leaching was reduced from 32.5 mg/L to 5.3 mg/L—approaching but still slightly above the 5 mg/L limit. Quantitatively, the removal rates for lead were 92.2%, 85.6%, 81.6%, and 84.6% at initial concentrations of 100, 300, 500, and 700 mg/kg, respectively; for zinc, the removal rates were 88.2%, 85%, 81.9%, and 84.4%. These results demonstrate that MICP technology effectively stabilizes lead and zinc ions in rare earth tailings, significantly reducing their environmental mobility.

### 5.5. Mineral composition analysis

Fig 13 presents the mineral composition of ion-adsorbed rare earth tailings soil after solidification/stabilization treatment under varying heavy metal concentrations, using cementation solution concentrations of 0.5 mol/L and 1.0 mol/L. The results indicated that calcite, quartz, vaterite, and metallic carbonate precipitates were consistently detected in the tailings soil treated by MICP solidification and stabilization. Regarding the mechanism of mineral formation, during the treatment process, calcium ions from the cementation solution ultimately precipitate as minerals such as calcite and quartz [59]. The formation of these precipitates is the direct factor responsible for the enhanced strength of the solidified tailings soil [60]. The mineral precipitates formed by calcium ions act as cementing and filling agents between tailings soil particles, strengthening the interparticle interactions and thereby improving the overall strength of the tailings soil. From the perspective of heavy metal stabilization, heavy metal ions such as lead and zinc in the rare earth tailings soil precipitate by forming coprecipitates with calcium carbonate and by combining with CO_3_^2−^ to generate heavy metal carbonates. The formation of these coprecipitates not only reduces the mobility of heavy metal ions within the tailings soil, mitigating their potential environmental hazards, but also imparts a skeleton-like role within the tailings soil structure, increasing its cohesion and resistance to deformation [58]. Therefore, the formation of heavy metal carbonates and their coprecipitation with calcium carbonate is a direct mechanism for heavy metal stabilization via MICP and is also one of the factors contributing to the improvement in strength of the tailings soil.

As shown in Fig 13, a comparison of the XRD patterns at cementation solution concentrations of 0.5 mol/L and 1.0 mol/L reveals distinct differences in diffraction peaks, indicating significant changes in the mineral composition of the soil samples. Specifically, the diffraction peak intensity corresponding to calcite in the 1.0 mol/L group is markedly higher than that in the 0.5 mol/L group, suggesting that a higher concentration of cementation solution facilitated the formation of more calcium carbonate precipitates. In addition, with the increase in the concentration of heavy metal contaminants in the soil, the amount of heavy metal carbonates formed increased significantly, while the content of calcium carbonate precipitates shows a negative correlation with the formation of metal carbonates. This result is consistent with the aforementioned findings.

### 5.6. Scanning electron microscopy analysis and solidification/stabilization mechanisms

**Fig 14:**
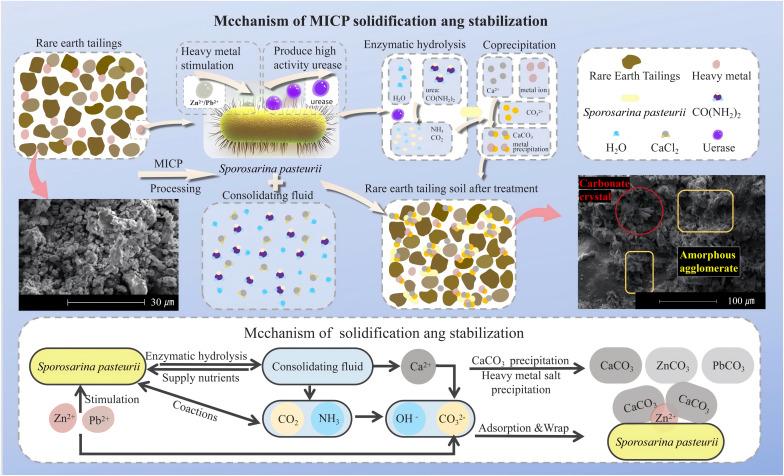
SEM image and mechanism analysis of microbial treatment of rare earth tailings.

Fig 14 presents the scanning electron microscopy images and mechanistic analysis of microbially treated rare earth tailings soil. Observations revealed the presence of calcium carbonate crystals deposited via microbial induction within the interparticle voids. The predominant morphology of these calcium carbonate crystals consisted of interlocked rhombohedra, with surfaces exhibiting numerous irregular aggregates. From a micromechanical perspective, these rough-surfaced calcite crystals significantly increase the surface roughness of soil particles, thereby enhancing the interparticle friction angle—a key factor contributing to the improved UCS values observed in Section 5.3 (up to 770 kPa at optimal conditions). Concurrently, the cementation effect exerted by these products effectively bonds the originally loose soil particles, ultimately leading to a substantial improvement in soil strength.

Furthermore, small clusters of lead mineral crystals and zinc mineral crystals, displaying a radiating orthorhombic crystal habit, were observed in localized regions. Between soil particles and calcite crystals, amorphous aggregates formed by the coprecipitation of calcium carbonate and carbonate crystals were also present. The formation process of these products was as follows: During the solidification/stabilization process employing cyclic grout injection, the bacterial solution is first thoroughly mixed with the heavy metal-containing rare earth tailings soil. In this stage, CO_3_^2−^ generated from urea hydrolysis by the bacteria preferentially reacts chemically with heavy metal ions in the tailings soil, forming carbonate crystallites. Subsequently, Ca^2+^ introduced via the cementation solution reacts with further CO_3_^2−^ produced by continued urea hydrolysis, generating abundant calcite crystals between soil particles. These calcite crystals predominantly encapsulate the previously formed carbonate crystallites. The aforementioned products fill the pores between soil particles.

Regarding the removal of heavy metals from soil, within the soil sample, some free heavy metal ions are adsorbed by calcium carbonate crystals and by aggregates formed on particle surfaces, thereby immobilizing them. Moreover, during the MICP solidification process, some heavy metal ions undergo chemical reactions to form heavy metal carbonate precipitates, which are also effectively immobilized. These combined mechanisms—adsorption and precipitation—collectively explain the significantly reduced leaching concentrations of Pb^2+^ and Zn^2+^ reported in Section 5.4, where removal rates reached up to 92.2% for lead and 88.2% for zinc. The visual evidence of heavy metal-containing precipitates and their association with calcite provides microstructural support for the stabilization efficacy demonstrated in the leaching tests.

### 5.7. Pearson correlation analysis

**Fig 15:**
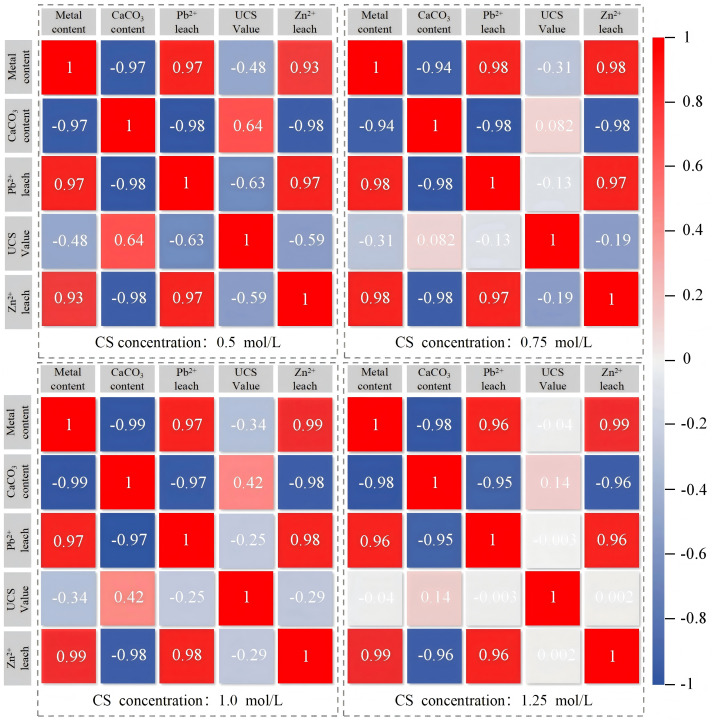
Pearson correlation analysis of MICP-treated rare earth tailings.

In the Pearson correlation equation (4), the value range of the correlation coefficient ‘r’ is [−1, 1], and its absolute value |r| directly quantifies the strength of the linear relationship between x and y. According to widely adopted threshold standards, |r| ≥ 0.8 indicates a high correlation, 0.5 ≤ |r| < 0.8 corresponds to a moderate correlation, 0.3 ≤ |r| < 0.5 is considered a weak correlation, and |r| < 0.3 indicates almost no correlation. The correlations of various parameters of ion-adsorbed rare earth tailing soils before and after MICP stabilization are shown in Fig 15.

For each cementation solution concentration, correlation analyses were based on n = 15 independent samples (five heavy metal concentration levels × three replicates), and the strong correlations (|r| > 0.8) were highly significant (p < 0.001). As shown in Fig 15, calcium carbonate/carbonate content exhibits a significant negative correlation with the initial heavy metal concentration in the soil samples. Notably, the degree of this negative correlation varies with cementation solution concentration: it strengthens as the concentration increases from 0.5 mol/L to 1.0 mol/L, but weakens at 1.25 mol/L. This variation reflects the dual effect of cementation solution concentration on MICP efficiency, with optimal performance observed at 1.0 mol/L.

The unconfined compressive strength (UCS) of the soil samples shows a weak negative correlation with initial heavy metal concentration and a weak positive correlation with calcium carbonate content. This relatively weak correlation can be attributed to the relationship between UCS and these two factors. Specifically, UCS initially increases with heavy metal concentration (from 0 to 300 mg/kg) but decreases at higher concentrations (500–700 mg/kg). Similarly, UCS increases with calcium carbonate content up to an optimal point, but declines when the cementation solution concentration is excessive, leading to uneven carbonate distribution and reduced total precipitation.

In contrast, the leaching concentrations of Pb^2+^ and Zn^2+^ show consistently strong negative correlations with calcium carbonate/carbonate content across all cementation solution concentrations (|r| > 0.8). This indicates that higher carbonate precipitation is associated with lower heavy metal leaching, further demonstrating that calcium carbonate yield serves as the material basis for the soil’s solidification/stabilization properties.

## 6. Conclusions

(1) The conventional mixing method had limited reinforcement effects on the rare earth tailings soil. A low-concentration cementation solution cannot effectively improve unconfined compressive strength (UCS), with only limited improvement observed at concentrations above 1.00 mol/L.
(2) The cyclic grout immersion method can significantly improve soil strength, with UCS showing a positive correlation with the content of generated calcium carbonate. At a cementation solution concentration of 1.00 mol/L, the UCS reaches a maximum of 770 kPa, with no strength reduction at excessively low concentrations.
(3) The initial heavy metal concentration in soil has a significant impact on the reinforcement effect: at low concentrations, it can be remediated by microorganisms, generating solid-phase minerals that strengthen the soil; at high concentrations, it suppresses bacterial activity, reduces calcium carbonate precipitation, and leads to a significant decrease in strength due to the increase in soluble salt content.
(4) This study innovatively revealed the high efficiency of the cyclic grout immersion method in the solidification/stabilization of rare earth tailings soil. Still, its practical application remains limited by heavy metal toxicity thresholds and bacterial tolerance.
(5) While this study demonstrates the efficacy of MICP for rare earth tailings solidification under controlled conditions, several practical challenges must be addressed before field application. These include the cost-effectiveness of large-scale bacterial culture and cementation solution preparation, the scalability of treatment duration given that field implementation typically requires longer processing timelines, and the long-term stability of bio-cemented tailings under environmental stressors such as wet-dry cycles, acid rain, and erosion. Future research should focus on optimizing these aspects to bridge the gap between laboratory findings and reliable field applications.

## References

[1] Huang X, Long Z, Wang L, Feng Z. Technology development for rare earth cleaner hydrometallurgy in China. Rare Metals. 2015;34(4):215–22. doi: 10.1007/s12598-015-0473-x

[2] Chun LZ, Huan LW, Wen JF, Sheng LZ, Xing JW, Jing S. Research progress on weathered crust elution-deposited rare earth ores. J China Univ Min Technol. 2022;51:1178–92. doi: 10.1007/s12598-022-01975-6

[3] Chen T, Wen X-C, Zhang L-J, Tu S-C, Zhang J-H, Sun R-N, et al. The geochemical and mineralogical controls on the release characteristics of potentially toxic elements from lead/zinc (Pb/Zn) mine tailings. Environ Pollut. 2022;315:120328. doi: 10.1016/j.envpol.2022.120328 36202267

[4] Zhang Z, Yang J, Guo W, Jiang L, Chen W, Liu D, et al. Analysis and prediction of the leaching process of ionic rare earth: a data mining study with scarce data. Minerals. 2024;14(6):596. doi: 10.3390/min14060596

[5] Wang GF, Xu J, Ran LY, Zhu RL, Ling B, Ling XL, et al. A green and efficient technology to recover rare earth elements from weathering crusts. Nat Sustain. 2023;6:81–92. doi: 10.1038/s41893-022-00989-3

[6] Peng J, Liu Z. Influence of temperature on microbially induced calcium carbonate precipitation for soil treatment. PLoS One. 2019;14(6):e0218396. doi: 10.1371/journal.pone.0218396 31211807 PMC6581288

[7] Migaszewski ZM, Gałuszka A, Dołęgowska S. Extreme enrichment of arsenic and rare earth elements in acid mine drainage: case study of Wiśniówka mining area (south-central Poland). Environ Pollut. 2019;244:898–906. doi: 10.1016/j.envpol.2018.10.106 30469284

[8] Wang X, Zhao C, Fan J, Zhao Q, Zhang X, Zhang N, et al. Contamination status and health risk assessment of potentially toxic trace elements in soils surrounding rare earth tailings in China: a retrospective review. Ecotoxicol Environ Saf. 2025;298:118270. doi: 10.1016/j.ecoenv.2025.118270 40334538

[9] Zheng X, Qiu S, Zhou B, Li Q, Chen M. Leaching of heavy metals from tungsten mining tailings: a case study based on static and kinetic leaching tests. Environ Pollut. 2024;342:123055. doi: 10.1016/j.envpol.2023.123055 38065334

[10] Zhang J, Li H, Ren G, Zhang Z, Nie S. Advancing sustainable mining practices: a dynamic ecological security and risk warning framework for ion-absorbed rare earth mine. J Clean Prod. 2025;511:145630. doi: 10.1016/j.jclepro.2025.145630

[11] Fan X, Xue Q, Liu S, Tang J, Qiao J, Huang Y, et al. The influence of soil particle size distribution and clay minerals on ammonium nitrogen in weathered crust elution-deposited rare earth tailing. Ecotoxicol Environ Saf. 2021;208:111663. doi: 10.1016/j.ecoenv.2020.111663 33396173

[12] Rajasekar A, Wilkinson S, Moy CKS. MICP as a potential sustainable technique to treat or entrap contaminants in the natural environment: a review. Environ Sci Ecotechnol. 2021;6:100096. doi: 10.1016/j.ese.2021.100096 36159179 PMC9488051

[13] Wang R, Tang C-S, Pan X, Shen Z, Liu Y, Lu X. A biotechnological approach for suspended solids removal in biogas slurry via microbially induced calcite precipitation (MICP). J Clean Prod. 2024;459:142537. doi: 10.1016/j.jclepro.2024.142537

[14] Cao HY, Gao GL, Rao LY, Zhang Y, Sun Z, Zhang JX. Microbially induced calcium carbonate precipitation to combat desertification: a field application experiment. J Clean Prod. 2024;468:143085. doi: 10.1016/j.jclepro.2024.143085

[15] Ahenkorah MMR, Karim MR, Beecham S. Unconfined compressive strength of MICP and EICP treated sands subjected to cycles of wetting-drying, freezing-thawing, and elevated temperature: experimental and EPR modeling. J Rock Mech Geotech Eng. 2023;15:1226–47. doi: 10.1016/j.jrmge.2022.12.004

[16] Liu HL, Chang Z, Yang X. Microbial mineralization: Reaction principles, deposition/destruction mechanisms, and theoretical frameworks: progress and challenges. Chin J Geotech Eng. 2024;46:1347–58.

[17] Liu H, Ma G, Zhao C, Zhang J, He X. Macro-micro mechanical mechanisms of microbially reinforced calcareous sand. J Civ Environ Eng. 2020;42:205–6. doi: 10.11835/j.issn.2096-6717.2020.038

[18] Zúñiga-Barra H, Velastegui E, Toledo-Alarcón J, Jorquera L, Rivas M, Jeison D. Investigation of the key factors favoring the bio-cementing effect of microbially induced calcite precipitation when applied to mine tailings. Appl Geochem. 2025;179:106258.

[19] Zhang BL, Yue J, Zhang HL, Zhao L, Kong Q, Gu L. A study on the mechanical properties of soil in the Yellow River flooding area improved by MICP technology. Build Sci. 2020;36:79–86. doi: 10.13614/j.cnki.11-1962/tu.2020.03.011

[20] Tao H, Sun C, Qu J, Huang Y. Study on the effect of cementation solution concentration on sand fixation by fiber reinforced MICP. PLoS One. 2025;20(8):e0329673. doi: 10.1371/journal.pone.0329673 40788957 PMC12338797

[21] Zhang Z, Tong K, Hu L, Yu Q, Wu L. Experimental study on solidification of tailings by MICP under the regulation of organic matrix. Constr Build Mater. 2020;265:120303. doi: 10.1016/j.conbuildmat.2020.120303

[22] Comadran-Casas C, Unluer C, Bass AM, Macdonald J, Khaksar Najafi E, Spruzeniece L, et al. Bioremediation of multiple heavy metals through biostimulation of microbial-induced calcite precipitation at varying calcium-to-urea concentrations. J Hazard Mater. 2025;491:137691. doi: 10.1016/j.jhazmat.2025.137691 40088671

[23] Achal V, Pan X, Zhang D, Fu Q. Bioremediation of Pb-contaminated soil based on microbially induced calcite precipitation. J Microbiol Biotechnol. 2012;22(2):244–7. doi: 10.4014/jmb.1108.08033 22370357

[24] Kumar A, Song H-W, Mishra S, Zhang W, Zhang Y-L, Zhang Q-R, et al. Application of microbial-induced carbonate precipitation (MICP) techniques to remove heavy metal in the natural environment: a critical review. Chemosphere. 2023;318:137894. doi: 10.1016/j.chemosphere.2023.137894 36657570

[25] Ji G, Huan C, Zeng Y, Lyu Q, Du Y, Liu Y, et al. Microbiologically induced calcite precipitation (MICP) in situ remediated heavy metal contamination in sludge nutrient soil. J Hazard Mater. 2024;473:134600. doi: 10.1016/j.jhazmat.2024.134600 38759409

[26] Sun Y, Zhong X, Lv J, Wang G. Experimental study on silt soil improved by microbial solidification with the use of lignin. Microorganisms. 2023;11(2):281. doi: 10.3390/microorganisms11020281 36838245 PMC9965713

[27] Zhuang D, Yao W, Guo Y, Chen Z, Gui H, Zhao Y. Bioremediation of heavy metal-contaminated solution and aged refuse by microbially induced calcium carbonate precipitation: further insights into *Sporosarcina pasteurii*. Microorganisms. 2025;13(1):64. doi: 10.3390/microorganisms13010064 39858832 PMC11767937

[28] Héctor ZB, Javiera TA, Álvaro TA, Lorena J, Mariella R, Leopoldo G, et al. Improving the sustainable management of mining tailings through microbially induced calcite precipitation: a review. Miner Eng. 2022;189:107855. doi: 10.1016/j.mineng.2022.107855

[29] Héctor ZB, Edgar V, Javiera TA, Loren J, Mariella R, David J. Investigation of the key factors favouring the bio-cementing effect of microbially induced calcite precipitation when applied to mine tailings. Appl Geochem 2025;179:106258.

[30] Jiang ZY, Chen Y, Fu JZ, Zhou H, Wen Z. Experimental study on remediation of cadmium-contaminated tailings via microbial-induced carbonate precipitation. Chin J Geotech Eng. 2025;47(6):1308–17. doi: 10.11779/j.issn.1000-4548.2024QY0010

[31] Lu T, Wei Z, Wang W, Yang Y, Cao G, Wang Y, et al. Experimental Investigation of sample preparation and grouting technology on microbially reinforced tailings. Constr Build Mater. 2021;312:125458. doi: 10.1016/j.conbuildmat.2021.125458

[32] Guo G, Zhou Q, Li X. Advances in research on in situ chemo-immobilization of heavy metals in contaminated soils. Ying Yong Sheng Tai Xue Bao. 2005;16(10):1990–6. 16422528

[33] Guan Y, Zhang N, Li B, Ma T, Wu W, Shi R. A novel evaluation of farmland soil environmental risk integrating heavy metal(loid) pollution risk and industrial risk potential. Stoch Environ Res Risk Assess. 2025;39(7):3085–102. doi: 10.1007/s00477-025-03010-3

[34] Taharia M, Dey D, Das K, Sukul U, Chen J-S, Banerjee P, et al. Microbial induced carbonate precipitation for remediation of heavy metals, ions and radioactive elements: a comprehensive exploration of prospective applications in water and soil treatment. Ecotoxicol Environ Saf. 2024;271:115990. doi: 10.1016/j.ecoenv.2024.115990 38262090

[35] Baziar MH, Alibolandi M. Liquefaction evaluation of microbial induced calcium carbonate precipitation (MICP) treated sands; a strain energy approach. J Earthq Eng. 2023;27(15):4512–25. doi: 10.1080/13632469.2023.2171508

[36] Su YL, Qu FL, Meng Y, Xu WJ, Zhu XH, Zhan C. Microbial-induced carbonate precipitation (MICP) modified biochar for low-carbon cementitious materials. Constr Build Mater. 2024;451:138644. doi: 10.1016/j.conbuildmat.2024.138644

[37] Muynck WD, Belie DN, Verstraete W. Microbial carbonate precipitation in construction materials: a review. Ecol Eng. 2010;36(2):118–36.

[38] Zhu X, Li W, Zhan L, Huang M, Zhang Q, Achal V. The large-scale process of microbial carbonate precipitation for nickel remediation from an industrial soil. Environ Pollut. 2016;219:149–55. doi: 10.1016/j.envpol.2016.10.047 27814530

[39] Kumar A, Song H-W, Mishra S, Zhang W, Zhang Y-L, Zhang Q-R, et al. Application of microbial-induced carbonate precipitation (MICP) techniques to remove heavy metal in the natural environment: a critical review. Chemosphere. 2023;318:137894. doi: 10.1016/j.chemosphere.2023.137894 36657570

[40] Peng J, Feng QP, Sun Y. Temperature effects on microbial-induced calcium carbonate precipitation for sand reinforcement. J Geotech Eng. 2018;40(6):1048–55.

[41] Rajasekar A, Wilkinson S, Moy CKS. MICP as a potential sustainable technique to treat or entrap contaminants in the natural environment: a review. Environ Sci Ecotechnol. 2021;6:100096. doi: 10.1016/j.ese.2021.100096 36159179 PMC9488051

[42] Gat D, Ronen Z, Tsesarsky M. Soil bacteria population dynamics following stimulation for ureolytic microbial-induced CaCO3 precipitation. Environ Sci Technol. 2016;50(2):616–24. doi: 10.1021/acs.est.5b04033 26689904

[43] Hu Q, Song W, Hu J. Study of the mechanical properties and water stability of microbially cured, coir-fiber-reinforced clay soil. Sustainability. 2023;15(17):13261. doi: 10.3390/su151713261

[44] Mujah D, Cheng L, Shahin MA. Microstructural and geomechanical study on biocemented sand for optimization of MICP process. J Mater Civil Eng. 2019;31(4):04019025. doi: 10.1061/(ASCE)MT.1943-5533.0002660

[45] Tserkis S, Assad SM, Lam PK, Narang P. Quantifying total correlations in quantum systems through the Pearson correlation coefficient. Phys Lett A. 2025;543:130432. doi: 10.1016/j.physleta.2025.130432

[46] Fu T, Saracho AC, Haigh SK. Microbially induced carbonate precipitation (MICP) for soil strengthening: a comprehensive review. Biogeotechnics. 2023;1(1):100002. doi: 10.1016/j.bgtech.2023.100002

[47] Zhu M, Wang W, Zhang T, Zou C, Zhang W, Zhao L, et al. One-step MICP-MAP solidification of rare earth slag enabled by a slow-release phosphorus source. J Environ Chem Eng. 2025;13(6):119615. doi: 10.1016/j.jece.2025.119615

[48] Yao D, Wu J, Wang G, Wang P, Zheng J-J, Yan J, et al. Effect of wool fiber addition on the reinforcement of loose sands by microbially induced carbonate precipitation (MICP): mechanical property and underlying mechanism. Acta Geotech. 2021;16(5):1401–16. doi: 10.1007/s11440-020-01112-6

[49] Wang S, Fang L, Dapaah MF, Niu Q, Cheng L. Bio-remediation of heavy metal-contaminated soil by microbial-induced carbonate precipitation (MICP)—a critical review. Sustainability. 2023;15(9):7622. doi: 10.3390/su15097622

[50] Tang C-S, Yin L, Jiang N, Zhu C, Zeng H, Li H, et al. Factors affecting the performance of microbial-induced carbonate precipitation (MICP) treated soil: a review. Environ Earth Sci. 2020;79(5). doi: 10.1007/s12665-020-8840-9

[51] Wang X, Zhang S, Chen K. Enhanced biogeochemical remediation of Pb-contaminated loess via MICP integrated with graphene nanomaterials. RSC Adv. 2025;15(35):29063–76. doi: 10.1039/d5ra04818d 40861985 PMC12377051

[52] Kapp S, Schmidt M, Streckfuss U, Müller R, Brenner-Weiß G. Inhibition of urease from *Sporosarcina pasteurii* by heavy metal ions: a kinetic and structural study. Sci Rep. 2020;10(1):18542. doi: 10.1038/s41598-020-75690-233122697

[53] Xie Y, Chen L, Liu W, Zhao Q, Zhang X, Wang H. Mechanisms of heavy metal toxicity on urease activity and biofilm formation in Sporosarcina pasteurii during MICP process. J Hazard Mater. 2024;465:133245. doi: 10.1016/j.jhazmat.2023.133245 38150761

[54] Hu J, Zhao Y, Chen X, Liu L, Wang Q. Tolerance and response of *Bacillus intermedius* TSBOI to lead and zinc stress during microbially induced carbonate precipitation. J Clean Prod. 2024;435:140567. doi: 10.1016/j.jclepro.2024.140567

[55] Jiang N-J, Liu R, Du Y-J, Bi Y-Z. Microbial induced carbonate precipitation for immobilizing Pb contaminants: Toxic effects on bacterial activity and immobilization efficiency. Sci Total Environ. 2019;672:722–31. doi: 10.1016/j.scitotenv.2019.03.294 30974362

[56] Zha F, Wang H, Kang B, Liu C, Xu L, Tan X. Improving the strength and leaching characteristics of Pb-contaminated silt through MICP. Crystals. 2021;11(11):1303. doi: 10.3390/cryst11111303

[57] Bhadiyadra SCJ, Ong DEL, Doh J-H. Trends and opportunities for greener and more efficient microbially induced calcite precipitation pathways: a strategic review. Geotech Res. 2024;11(3):161–85. doi: 10.1680/jgere.24.00039

[58] Lu T, Wei ZA, El Naggar MH, Wang WS, Yang YH, Tian X, et al. Effect of chemical environment on copper tailings reinforced by microbially induced carbonate precipitation. Constr Build Mater. 2023;400:132894. doi: 10.1016/j.conbuildmat.2023.132894

[59] Payan M, Sangdeh MK, Salimi M, Ranjbar PZ, Arabani M, Hosseinpour I. A comprehensive review on the application of microbially induced calcite precipitation (MICP) technique in soil erosion mitigation as a sustainable and environmentally friendly approach. Results Eng. 2024;24:103235. doi: 10.1016/j.rineng.2024.103235

[60] Song H-W, Li D, Qiu H, Yu Z-G, Kumar A, Yan X-X, et al. Microbial-induced carbonate precipitation effectively prevents Pb2+ migration through the soil profile: Lab experiment and model simulation. Sci Total Environ. 2024;927:172268. doi: 10.1016/j.scitotenv.2024.172268 38583629

